# Prospective associations between the CKD-mineral bone disorder and metabolic acidosis

**DOI:** 10.1007/s11255-025-04902-7

**Published:** 2025-12-18

**Authors:** Hulya Taskapan, Sara Mahdavi, Antonio Bellasi, Berkay Taskapan, Paul Tam, Tabo Sikaneta

**Affiliations:** 1 Kidney Life Sciences Institute, Toronto, Canada; 2 Scarborough Health Network, Toronto, Canada; 3 University of Toronto, Toronto, Canada; 4 Department of Nephrology, Ente Ospedaliere Cantonale, Lugano, Switzerland

**Keywords:** Chronic kidney disease, Bone mineral disorder of CKD, Metabolic acidosis, Subclinical metabolic acidosis, Hyperparathyroidism, Vitamin D deficiency, Hyperphosphatemia, Hyperchloremia, Hypocalcemia, FGF-23

## Abstract

**Background:**

The common chronic kidney disease (CKD)-associated complications of mineral bone disorder (MBD) and metabolic acidosis share some biochemical features that physiologic studies suggest may be linked by the effects of progressive acidosis on bone and kidney. To explore this further, we examined prospective associations between key elements of the CKD-MBD and the subsequent appearance of subclinical and clinical metabolic acidosis in patients with stage 3/4 CKD.

**Methods:**

Post hoc analysis of 1993 CAN AIM TO PREVENT participants followed for 2.86 years per participant. Mixed-effects linear and logistic regression were used to evaluate prospective associations between phosphate, calcium, 25(OH)D, PTH, and FGF-23 with (1) declining plasma bicarbonate, (2) subclinical metabolic acidosis (bicarbonate 22–24 mmol/L), and (3) clinical metabolic acidosis (bicarbonate < 22 mmol/L).

**Results:**

Each mmol/L increase in phosphate predicted a lower bicarbonate (− 1.29 mmol/L (95% CI − 1.61, − 0.98; *p* < 0.001)), and increased odds for subclinical (OR 1.92 (1.32, 2.79); *p* = 0.001) and clinical metabolic acidosis (OR 5.03 (3.16, 7.93); *p* < 0.001). Each mmol/L increase in calcium predicted a higher bicarbonate (2.70 mmol/L (2.11, 3.30; *p* < 0.001)), and decreased odds for subclinical (OR 0.23 (0.11, 0.46); *p* < 0.001) and clinical metabolic acidosis (OR 0.15 (0.06, 0.37); *p* < 0.001). Each unit increase in log-transformed 25(OH)D predicted a higher bicarbonate (0.38 mmol/L (0.23, 0.53); *p* < 0.001)), and decreased odds for subclinical (OR 0.67 (0.56, 0.80); *p* < 0.001) and clinical metabolic acidosis (OR 0.65 (0.50, 0.82); *p* < 0.001). Log-PTH exhibited a non-linear relationship with acidosis risk: moderate elevations were associated with reduced acidosis risk (OR 0.60 (0.40, 0.90); *p* = 0.013), while higher levels predicted an increased risk for subclinical (OR 1.42 (1.02, 1.98); *p* = 0.041) but not clinical metabolic acidosis (OR 1.44 (0.93, 2.24); *p* = 0.103). No significant associations were found between log-FGF-23 and acidosis risk (OR 1.02 (0.88, 1.19); *p* = 0.767).

**Conclusion:**

Key elements of the CKD-MBD were independently and prospectively associated with the subsequent development of subclinical and clinical metabolic acidosis. Future studies could examine whether a causal relationship exists between progressive metabolic acidosis and the CKD-MBD.

**Supplementary Information:**

The online version contains supplementary material available at 10.1007/s11255-025-04902-7.

## Introduction

Metabolic acidosis, defined as a bicarbonate less than 22 mmol/L, develops in 2–37% of patients with stage 3 and 4 chronic kidney disease (CKD) [1]. It contributes to the progression of CKD by promoting renal inflammation and fibrosis, exacerbates insulin resistance and related complications, induces muscle wasting, and impairs physical function and quality of life even when subclinical or before bicarbonate has declined to this level [2–4]. It arises when declining renal ammoniagenesis and proton excretion rates fail to clear, and intracellular and extracellular physiologic mechanisms fail to buffer daily net positive acid loads from endogenous and exogenous sources [5].

Among the buffering mechanisms, acid buffering by bone plays a prominent role, releasing sodium, potassium, phosphate, calcium, carbonate, and citrate in exchange for absorbing excess acid loads [2, 3, 5]. Metabolic acidosis also inhibits tubular resorption of filtered calcium, and thus hypercalciuria and negative calcium balance are consistently seen, despite acid-induced release of calcium from bone [6–11]. Metabolic acidosis impairs phosphate handling by reducing proximal tubular sodium-phosphate cotransporter activity, leading to elevated urinary phosphate excretion [12]. It also stimulates phosphate-regulating hormones such as fibroblast growth factor 23 (FGF-23) and parathyroid hormone (PTH) [11–13]. Both hyperphosphatemia and hypocalcemia promote changes in bone mineral metabolism—including elevations in fibroblast growth factor 23 (FGF-23), alterations in parathyroid hormone (PTH), and suppression of 1,25-hydroxyvitamin D (1,25-(OH)_2_D)—which collectively constitute the bone mineral disorder of CKD (CKD-MBD).

These physiologic observations support the premise that progressive metabolic acidosis and mineral bone disorder may be linked beyond sharing CKD as a predominant risk factor. Here we report on prospective associations between phosphate, calcium, 25-OH vitamin D (the substrate for 1,25-(OH)_2_D), PTH, and FGF-23 with declines in plasma bicarbonate and the development of subclinical and clinical metabolic acidosis in 1993 multi-ethnic patients with non-dialysis CKD.

## Materials and methods

### Study population, variables, and outcome definitions

This was a post hoc analysis of participants in the CAN AIM TO PREVENT (ClinicalTrials.gov: NCT01974713) [14], a prospective observational cohort study conducted between 2010 and 2015 at three non-dialysis CKD clinics in Toronto, Canada. All participants provided written informed consent, and the study protocol was approved by the institutional review boards of participating centers and conducted in accordance with the Declaration of Helsinki. The original trial enrolled 2,254 adult patients with CKD who were not receiving dialysis or renal transplantation. Participants for this analysis had a study entry eGFR between 15 and 60 ml/min/1.73 m^2^.

Baseline data collection encompassed demographic information, clinical history, medications, vital signs, and comprehensive laboratory measurements. Clinical and biochemical data collections were repeated at 6-month intervals over a 3-year follow-up period. The biochemical parameters analyzed in this study included bicarbonate, calcium, potassium, chloride, phosphate, albumin, FGF-23, 25(OH)D, and PTH that were measured using standardized laboratory techniques at each participating center. Additional potential confounders examined included age, sex, eGFR (using the 2009 CKD EPI equation), diabetes status, and bicarbonate supplementation, and were selected based on their established or theoretical relationships with acid–base homeostasis in CKD. PTH, FGF-23, and 25(OH)D were log-transformed to approximate normality.

Three primary outcomes were assessed: (1) plasma bicarbonate as a continuous variable; (2) subclinical metabolic acidosis (defined as bicarbonate between 22 and 24 mmol/L) and compared with bicarbonate > 24 mmol/L; and (3) clinical metabolic acidosis (defined as bicarbonate < 22 mmol/L) and compared with bicarbonate ≥ 22 mmol/L.

### Statistical analysis

All statistical analyses were performed using Stata Statistical Software, Release 18 (StataCorp, College Station, TX). Statistical significance was set at a two-tailed *p* value of < 0.05. Continuous variables were summarized as means ± standard deviations. Categorical variables were reported as frequencies and percentages. Between-group comparisons of baseline characteristics utilized independent samples *t *tests or Wilcoxon rank-sum tests for continuous variables and Chi-square tests for categorical variables.

Predictors of bicarbonate levels were analyzed using linear mixed-effects regression models, and subclinical and clinical metabolic acidosis using logistic mixed-effects regression models. These models analyze data with both fixed and unmodifiable factors (like age) and random factors (like individual variations) over time [15]. All models incorporated random intercepts and random slopes for visit number to capture individual-specific trajectories of bicarbonate change over time. A flexible unstructured covariance matrix was specified for the random effects to allow for distinct variances and covariances between the random intercept and random slope, optimizing the modeling of within-subject correlation. Robust variance estimation was employed to address potential heteroskedasticity and within-cluster correlation. This helps account for situations where the data may not follow perfect patterns or have some hidden relationships between grouped data points. All models were adjusted for age, sex, eGFR, diabetes mellitus, potassium, chloride, albumin, bicarbonate use, and visit time. We accounted for potential non-linear relationships using restricted cubic splines for log-transformed PTH and included a quadratic term for visit number to capture non-linear temporal trends. Restricted cubic splines are a technique for modeling data with curves instead of straight lines. They enable the data to bend and change direction at specific points, making the model more adaptable. This approach helps to capture complex relationships in the data without imposing a straight-line assumption [16].

No imputation was conducted as the analysis using mixed models minimized the impact of any missing values.

Results from linear regression models were expressed as changes in bicarbonate (in mmol/L), while logistic regression model outcomes were reported as odds ratios for the development of subclinical or clinical metabolic acidosis. Logistic model performance was evaluated using the area under the ROC curve (AUC). To ensure robust findings, we performed internal validation through bootstrap resampling with 1000 replicates, to confirm the stability of our estimates. Bootstrap analysis is a technique used to assess the reliability of a model by repeatedly sampling from the original dataset with replacement. It generates multiple “new” datasets by randomly selecting data points, allowing the model to be tested on each sample. This process helps evaluate the stability of the results, providing insight into how consistent or reliable the model’s conclusions are by comparing outcomes across many different resampled datasets [17].

## Results

### Study participants

Of the 2,254 CAN AIM TO PREVENT participants, 1,993 had stage 3 or 4 CKD at study entry and were analyzed in the current study. They had 11,395 study visits for an average of 5.7 visits and observation period of 2.86 years per participant. There were at least 11,291 measurements of bicarbonate and each examined CKD-MBD marker (missing values less than 1% per variable).

### Unadjusted characteristics by acidosis status at study baseline

Mean bicarbonate was 25.28 ± 3.27 mmol/L, and subclinical and clinical metabolic acidosis were present in 27.09% and 11.7%, respectively, at study entry. Comparisons by acidosis status showed that patients with clinical metabolic acidosis had higher chloride, anion gap, FGF-23, PTH, and lower calcium and 25(OH)D (*p* < 0.001 for all) (Table 1).

**Table 1:** Unadjusted characteristics by acidosis status at study baseline

Variables at baseline	All participants (*N* = 1993)	No metabolic acidosis (bicarbonate > 24) (*N* = 1220)	Subclinical metabolic acidosis (bicarbonate > 22–24) (*N* = 540)	*p*	No clinical metabolic acidosis (bicarbonate ≥ 22 mmol/L) (*N* = 1760)	Clinical metabolic acidosis (bicarbonate < 22 mmol/L) (*N* = 233)	*p*
	(Mean ± SD) or median (IQR)	(Mean ± SD) or median (IQR)	(Mean ± SD) or median (IQR)		(Mean ± SD) or median (IQR)	(Mean ± SD) or median (IQR)	
Bicarbonate (mmol/L)	25.28 ± 3.27	27.38 ± 1.93	23.21 ± 0.84	< 0.001	26.07 ± 2.55	19.42 ± 1.65	< 0.001
Age (year)	69.52 ± 12.02	70.11 ± 11.75	68.99 ± 12.18	0.0680	69.77 ± 11.89	67.64 ± 12.92	0.011
Male sex (%)	66.23%	65.16%	69.81%	0.056	66.59%	63.52%	0.352
Diabetes mellitus (%)	48,62%	46.72%	51.30%	0.076	48.13%	52.36%	0.224
eGFR (mL/min/1.73 m^2^)	38.53 ± 11.22	40.90 ± 10.42	36.18 ± 11.12	< 0.001	39.45 ± 10.85	31.54 ± 11.45	< 0.001
Chloride (mmol/L)	102.76 ± 3.51	101.86 ± 3.23	103.72 ± 3.28	< 0.001	102.44 ± 3.36	105.23 ± 3.65	< 0.001
Anion gap	11.94 ± 2.89	10.92 ± 2.23	12.80 ± 2.55	< 0.001	11.51 ± 2.49	15.11 ± 3.56	< 0.001
Potassium (mmol/L)	4.53 ± 0.56	4.41 ± 0.51	4.66 ± 0.54	< 0.001	4.48 ± 0.53	4.83 ± 0.62	< 0.001
PO4 (mmol/L)	1.17 ± 0.20	1.15 ± 0.18	1.17 ± 0.20	0.032	1.16 ± 0.19	1.25 ± 0.29	< 0.001
Calcium (mmol/L)	2.37 ± 0.12	2.38 ± 0.11	2.37 ± 0.12	0.0857	2.37 ± 0.12	2.33 ± 0.13	< 0.001
25(OH)D (nmol/L)	66 [45, 85]	67 [47, 87]	62 [40, 80]	0.0001	66 [45, 85]	61 [38, 81]	0.0122
PTH (pmol/L)	5.3 [3.6, 8.1]	4.9 [3.4, 7.4]	5.9 [3.8, 8.7]	< 0.001	5.2 [3.6, 7.8]	6.65 [4.6, 10.0]	< 0.001
FGF-23 (RU/mL)	127.3 [88.9, 179.4]	122.45 [84.9, 169.98]	134.9 [97.3, 191.4]	< 0.001	126 [87.8, 177.42]	140.1 [99.1, 192.7]	0.023
Albumin (g/L)	43.29 ± 3.19	43.40 ± 2.95	43.14 ± 3.49	0.1114	43.32 ± 3.13	43.01 ± 3.60	0.1567

### Adjusted changes in CKD-MBD elements in relation to risk for metabolic acidosis (Table 2, Figs. 1, 2, 3, 4, 5)

**Fig. 1:**
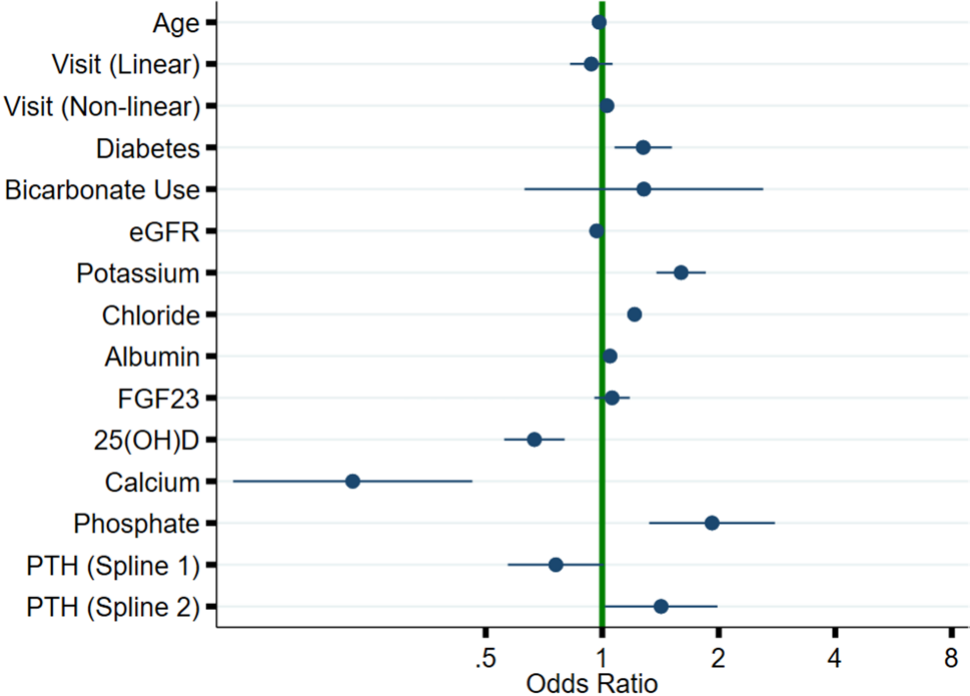
Predictors of subclinical metabolic acidosis

**Fig. 2:**
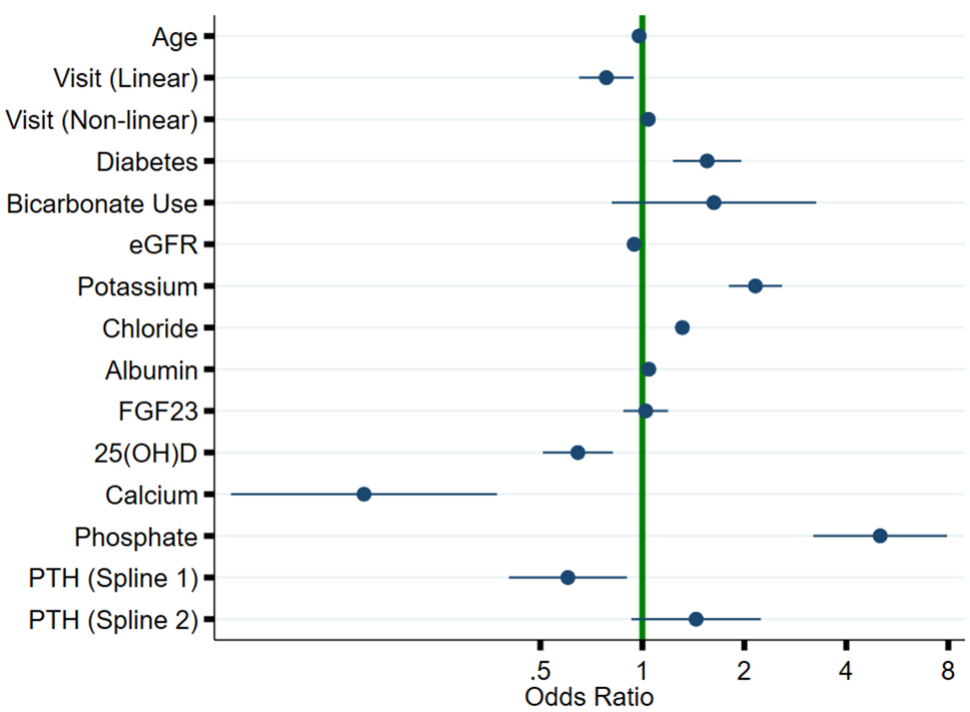
Predictors of clinical metabolic acidosis

**Fig. 3:**
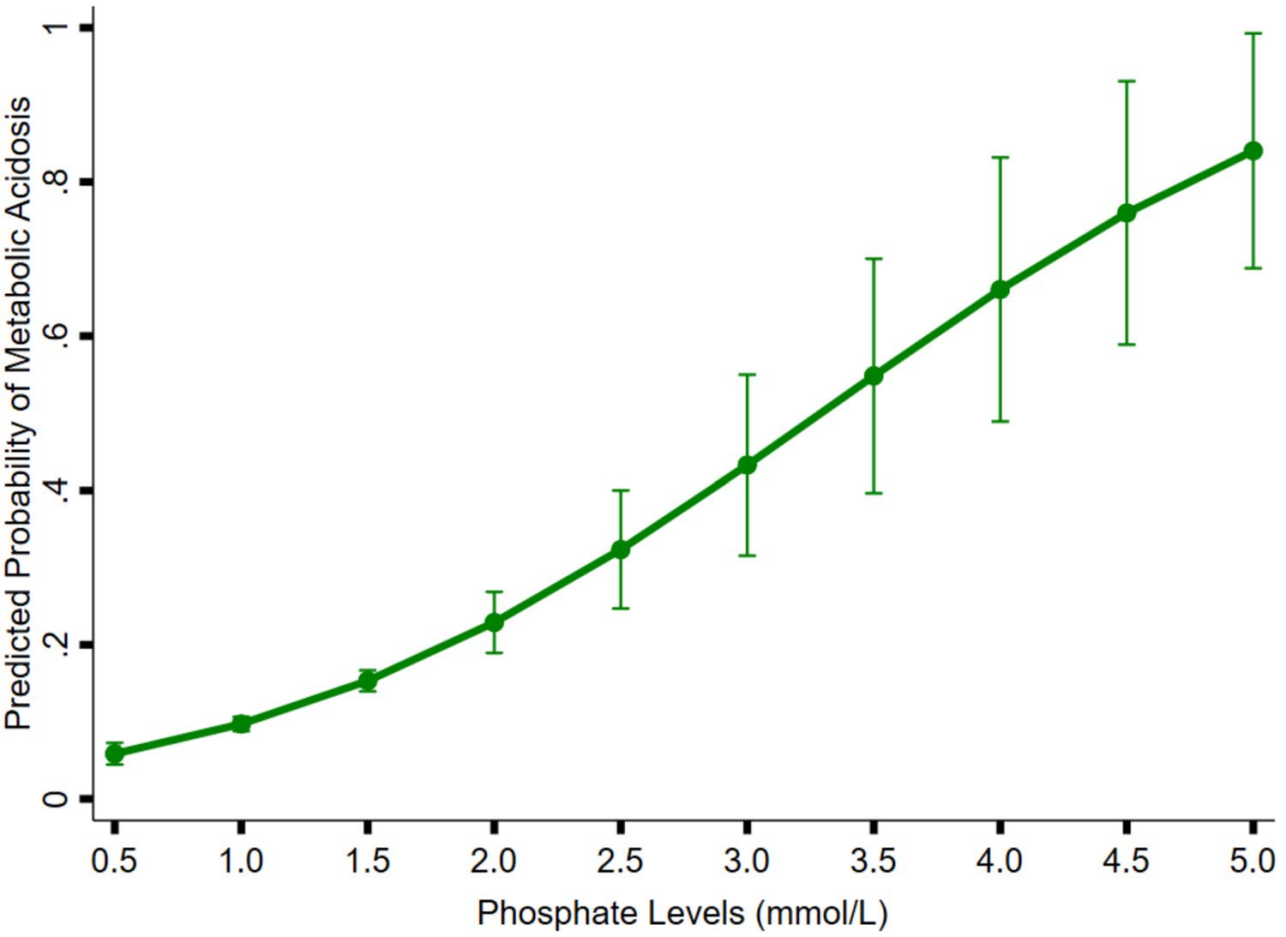
Predicted probability of metabolic acidosis as a function of phosphate levels

Phosphate: Each 1 mmol/L increase in phosphate predicted a lower bicarbonate (− 1.29 mmol/L (95% CI − 1.61, − 0.98; *p* < 0.001)), and increased odds for subclinical (OR 1.92; 95% CI 1.32, 2.79; *p* = 0.001) and clinical metabolic acidosis (OR 5.03; 95% CI 3.16, 7.93; *p* < 0.001) (Table 2 and Figs.1, 2, 3).

**Table 2:** Predictors of risk for metabolic acidosis

Variable	Change in plasma bicarbonate	Subclinical metabolic acidosis	Clinical metabolic acidosis
	per mmol/L, (95% CI), *p*	OR, (95% CI), *p*	OR, (95% CI), *p*
Age (per year)	0.02 (0.02, 0.03) < 0.001	0.98 (0.97, 0.99) < 0.001	0.98 (0.97, 0.99) < 0.001
Time (per 6 months)	0.08 (− 0.01, 0.18) 0.093	0.94 (0.82, 1.06) 0.314	0.78 (0.65, 0.94) 0.010
Time (non-linear)	− 0.03 (− 0.04, − 0.02) < 0.001	1.02 (1.01, 1.04) < 0.001	1.04 (1.01, 1.06) < 0.001
Diabetes mellitus (yes)	− 0.39 (− 0.57, − 0.22) < 0.001	1.28 (1.23, 1.96) 0.005	1.55 (1.23, 1.96) < 0.001
Sodium bicarbonate use (yes)	− 0.24 (− 0.79, 0.30) 0.390	1.28 (0.63, 2.61) 0.494	1.62 (0.81, 3.26) 0.170
eGFR (mL/min/1.73 m^2^)	0.05 (0.04, 0.06) < 0.001	0.97 (0.96, 0.98) < 0.001	0.95 (0.94, 0.96) < 0.001
Potassium (mmol/L)	− 0.56 (− 0.69, − 0.43) < 0.001	1.60 (1.38, 1.85) < 0.001	2.16 (1.80, 2.58) < 0.001
Log FGF-23	0.07 (− 0.01, 0.15) 0.105	1.06 (0.95, 1.18) 0.275	1.02 (0.88, 1.19) 0.767
Log-PTH (spline 1)	0.26 (0.02, 0.50) 0.035	0.76 (0.57, 1.01) 0.058	0.60 (0.40, 0.90) 0.013
Log-PTH (spline 2)	− 0.25 (− 0.53, 0.02) 0.074	1.42 (1.02, 1.98) 0.041	1.44 (0.93, 2.24) 0.103
Log 25(OH)D	0.38 (0.23, 0.53) < 0.001	0.67 (0.56, 0.80 < 0.001	0.65 (0.50, 0.82) < 0.001
Albumin (g/L)	− 0.07 (− 0.09, − 0.04) < 0.001	1.05 (1.02, 1.08) 0.001	1.04 (1.00, 1.08) 0.017
Phosphate (mmol/L)	− 1.29 (− 1.61, − 0.98) < 0.001	1.92 (1.32, 2.79) 0.001	5.03 (3.16, 7.93) < 0.001
Chloride (mmol/L)	− 0.26 (− 0.29, − 0.24) < 0.001	1.21 (1.18, 1.24) < 0.001	1.31 (1.27, 1.36) < 0.001
Calcium (mmol/L)	2.70 (2.11, 3.30) < 0.001	0.23 (0.11, 0.46) < 0.001	0.15 (0.06, 0.37) < 0.001

**Fig. 4:**
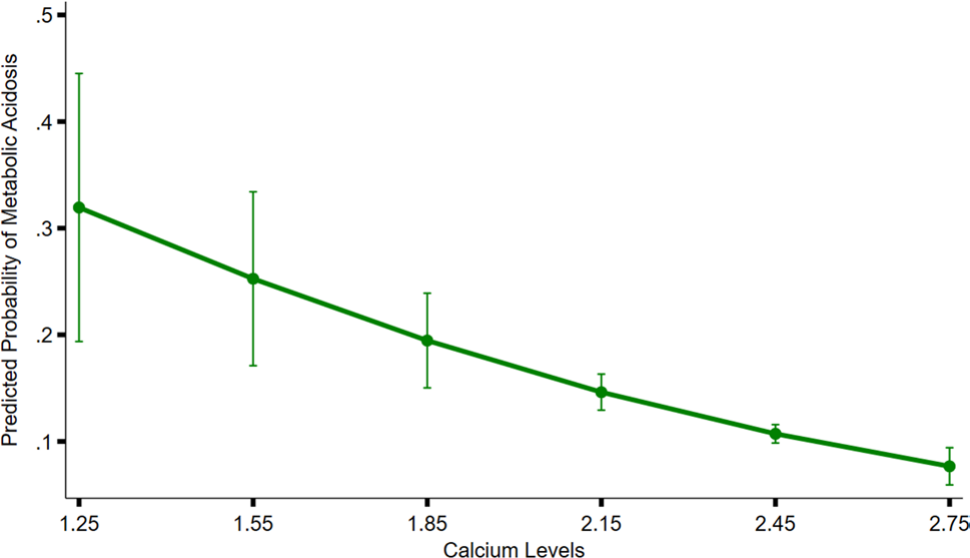
Predicted probability of metabolic acidosis as a function of calcium levels

Calcium: Each 1 mmol/L increase in calcium predicted a higher bicarbonate (2.70 mmol/L (95% CI 2.11, 3.30; *p* < 0.001)), and decreased odds for subclinical (OR 0.23; 95% CI 0.11, 0.46; *p* < 0.001) and clinical metabolic acidosis (OR 0.15; 95% CI 0.06, 0.37; *p* < 0.001) (Table 2 and Figs.1, 2 and 4).

25 OHD: Each unit increase in log-transformed 25(OH)D predicted a higher bicarbonate (0.38 mmol/L; 95% CI 0.23, 0.53; *p* < 0.001), and decreased odds for subclinical (OR 0.67; 95% CI 0.56, 0.80; *p* < 0.001) and clinical metabolic acidosis (OR 0.65; 95% CI 0.50, 0.82; *p* < 0.001).

**Fig. 5:**
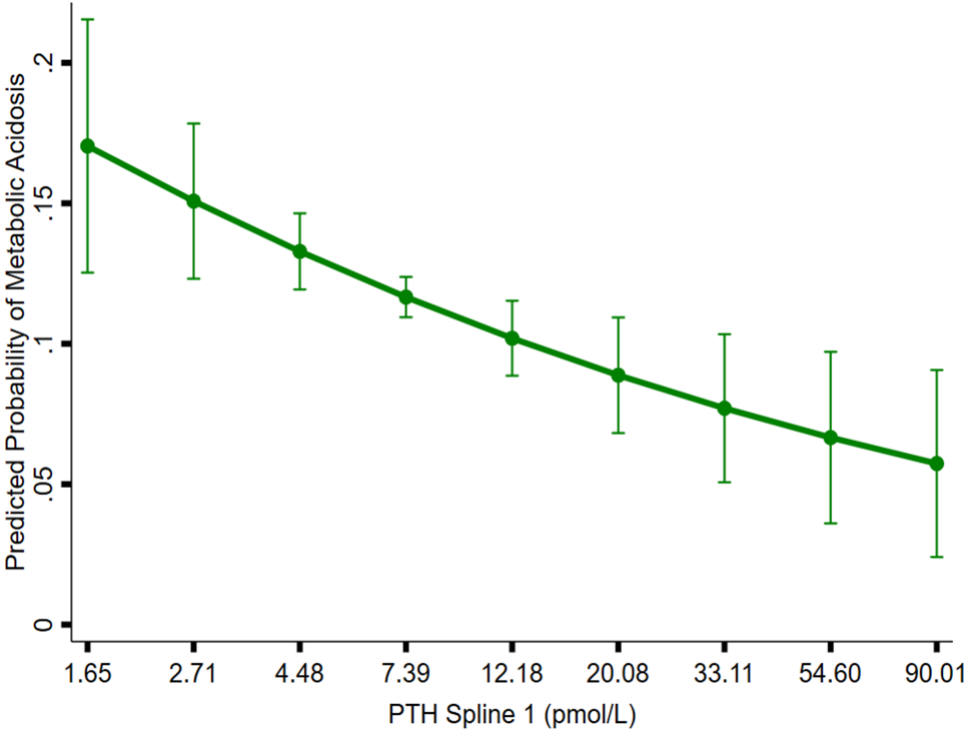
Predicted probability of metabolic acidosis as a function of the first spline of PTH

PTH: Log-transformed PTH exhibited a non-linear relationship with acidosis risk. Moderate elevations of PTH (log PTH spline 1) were associated with higher bicarbonate (0.26 mmol/L; 95% CI 0.02, 0.50; *p* = 0.035) and a reduced risk for both clinical (OR 0.60; 95% CI 0.40, 0.90; *p* = 0.013) (Fig. 5) and subclinical (OR 0.76; 95% CI 0.57, 1.01; *p* = 0.058) metabolic acidosis. However, at higher PTH levels (log PTH spline 2), bicarbonate predicted − 0.25 mmol/L (95% CI − 0.53, 0.02; *p* = 0.074), and this protective effect diminished, becoming non-significant for clinical acidosis (OR 1.44; 95% CI 0.93, 2.24; *p* = 0.103) but showing an increased risk for subclinical metabolic acidosis (OR 1.42; 95% CI 1.02, 1.98; *p* = 0.041) (Table 2 and Figs. 1, 2 and 5).

FGF-23: No significant association was found between log FGF-23 levels and bicarbonate concentrations (0.07 mmol/L; 95% CI − 0.01, 0.15; *p* = 0.105) or the odds for clinical (OR 1.02; 95% CI 0.88, 1.19; *p* = 0.767) or subclinical metabolic acidosis (OR 1.06; 95% CI 0.95, 1.18; *p* = 0.275).

Older age, history of diabetes, eGFR, potassium, chloride, and albumin were each also predictive of changes in bicarbonate levels and the odds of subclinical and clinical metabolic acidosis, as detailed in Table 2.

### Logistic model performance and robustness

Models demonstrated good discrimination (AUC = 0.88) for subclinical metabolic acidosis, and high discrimination (AUC > 0.94) for clinical metabolic acidosis (Supplemental Figs. 1 and 2). Bootstrap analysis (Supplement Table 1) validated the robustness of the identified predictors.

## Discussion

This study demonstrated that abnormalities in phosphate, calcium, 25(OH)D, and PTH consistent with those observed in the CKD-MBD were each prospectively associated with declining serum bicarbonate and the development of metabolic acidosis in patients with stage 3–4 CKD. The demonstration of temporal links between these key elements of the CKD-MBD and progressive acid loading suggests a greater degree of overlap than has previously been recognized. Future studies could validate these findings in other CKD populations, evaluate possible mechanisms such as the bone and kidney effects of metabolic acidosis, and assess for a possible causal association between progressive metabolic acidosis and the development of the CKD-MBD.

Phosphate serves as a crucial buffer against metabolic acidosis, contributing to acid–base homeostasis through multiple mechanisms [18]. Previous studies demonstrate increased urinary phosphorus excretion in response to acid loads in healthy individuals and animal model [7, 19]. However, in CKD, this relationship appears reversed, with Khairallah et al. [12] reporting higher phosphorus associated with greater acid loads. The molecular mechanisms underlying this altered relationship involve disrupted osteocyte signaling pathways, where metabolic acidosis directly stimulates FGF-23 production [13] and augments PTH levels through altered pH-dependent responsiveness of the calcium-sensing receptor [11, 20]. In addition, acidosis reduces proximal tubular sodium-phosphate cotransporter activity, impairing renal phosphate reabsorption and contributing to both increased serum phosphate and phosphaturia [11]. Clinical evidence supports this mechanism, as patients with serum bicarbonate < 22 mEq/L demonstrate 26% higher PTH levels and increased titratable acid excretion through monovalent phosphate [12].

While these studies focused on acid’s effects on phosphate metabolism, our findings provide a complementary perspective by demonstrating phosphate’s predictive role in metabolic acidosis. The relationship between phosphate and metabolic acidosis is complex and bidirectional. While the established literature demonstrates that acidosis influences phosphate homeostasis—through effects on renal handling and bone metabolism [12, 18, 21–24]—our findings highlight that elevated phosphate might also reflect increasingly strained acid-buffering mechanisms: persistently rising phosphate levels in the face of dietary restriction and phosphate-binding could reflect both impaired renal excretion and increased acid-buffering by bone. Future studies could evaluate if diet- and phosphate binder-independent hyperphosphatemia acts as a sentinel marker of, and is a potential mediator of adverse outcomes attributed to, metabolic acidosis in CKD patients.

Calcium is tightly regulated by its absorption across the small intestine, release from bone, and excretion by kidneys, and the metabolic acidosis of CKD can alter each of these [6–10]. For example, by suppressing 1-α hydroxylase, metabolic acidosis has been linked to reduced calcitriol-mediated intestinal calcium absorption in some but not all studies [9, 25]. Acid buffering by bone leads to dissolution of bone mineral matrix, releasing calcium and other bone mineral matrix elements, while chronic acidosis further exacerbates bone mineral release by suppressing osteoblast activity and increasing osteoclast activity [25, 26]. Metabolic acidosis also reduces passive paracellular resorption of filtered calcium in the proximal tubule and thick ascending limb through claudin-2 and claudin-16/19 channels, respectively, and reduces pH-dependent active transcellular resorption in the distal convoluted tubule through TRPV5 channels [8,23, 27]. Thus acidosis consistently increases urinary calcium excretion and lowers calcium in CKD patients who make urine [6]. Collectively these changes can lead to a negative calcium balance, have serious adverse consequences for bone health, and increase risk for calcium stone formation [1, 5–7, 26]. Future studies could confirm whether metabolic acidosis-induced calciuria is a cause of calcitriol-independent hypocalcemia in non-dialysis CKD patients.

Vitamin D influences acid–base homeostasis through multiple renal and extrarenal mechanisms [28, 29]. Early studies demonstrated that vitamin D deficiency impairs renal acidification, leading to elevated urinary pH despite normal hydrogen ion excretion. Whitten’s landmark studies revealed that vitamin D-deficient patients exhibit subclinical acidification defects, characterized by minimal tubular dysfunction and increased urinary excretion of basic amino acids and peptides [30]. Case reports of Fanconi syndrome demonstrated rapid reversal of metabolic acidosis and renal tubular defects following vitamin D supplementation [31]. Experimental studies in vitamin D-deficient chicks showed correction of hyperchloremic metabolic acidosis within 24 h of vitamin D administration, suggesting direct systemic effects on acid–base regulation independent of mineral metabolism [32]. Our findings extend this literature, demonstrating that vitamin D inadequacy (< 75 nmol/L) significantly increases the odds of metabolic acidosis in CKD patients. The protective effect of vitamin D likely involves enhancement of renal acidification processes, modulation of bone buffering capacity, and regulation of acid–base transporter expression. The results suggest that future studies concerned with correcting metabolic acidosis could consider evaluating the impact of restoring vitamin D to levels greater than 75 nmol/L.

PTH’s roles in acid–base regulation are complex. For example, its impact varies with exposure duration. Acutely, it inhibits proximal tubular bicarbonate reabsorption, while enhancing skeletal alkali mobilization and paradoxically increasing renal net acid excretion despite impaired bicarbonate reabsorption [33].  Increased urinary bicarbonate loss with PTH likely reflects higher filtered bicarbonate load from elevated levels [34, 35]. Chronic PTH exposure induces metabolic alkalosis in animal models with normal renal function [36–39], involving both renal and extrarenal mechanisms. Prolonged PTH administration maintains elevated bicarbonate even after net acid excretion normalizes [36, 37], and is accompanied by significant extrarenal bicarbonate generation [36, 37, 40, 41]. PTH-induced hypercalcemia further contributes to metabolic alkalosis by stimulating bicarbonate retention [38, 42]. The current study revealed a non-linear relationship between PTH and metabolic acidosis in CKD patients. We surmise that moderate elevations in PTH contribute to maintaining bicarbonate levels by enhancing bone alkali mobilization, and that this is no longer effective at higher elevations in PTH.

FGF-23, which is central to phosphate metabolism and often elevated in CKD as part of the CKD-MBD, could theoretically affect acid–base homeostasis via suppression of calcitriol synthesis, thus exacerbating vitamin D deficiency, hypocalcemia, and secondary hyperparathyroidism. However, we found no evidence of an independent association between FGF-23 and bicarbonate concentrations. One possible explanation is that any association of FGF-23 with metabolic acidosis risk is indirect or overshadowed by stronger predictors like phosphate, and PTH. Another possible explanation is that hypocalcemia reduces calcium-binding within the FGF-23 promoter, thus downregulating FGF-23 production. Observations that hyperphosphatemia fails to increase FGF-23 when hypocalcemia is present support this premise [43].

### Strengths and limitations

This study leveraged a large, well-characterized prospective CKD cohort with many routinely collected longitudinal follow-up data collected over a period of 3 years to enable robust analysis of temporal associations. The comprehensive dataset included detailed bone mineral metabolism parameters and repeated measurements that strengthened the results’ reliability. The inclusion of multiple centers (representing a diverse population) adds robustness to the conclusions. Our analysis extended to determining changes in bicarbonate before the development of clinical metabolic acidosis, adding new information about the biochemical predictors of subclinical metabolic acidosis.

Several limitations warrant consideration. As a post hoc analysis, this study was not designed to assess the associations between CKD-MBD and progressive metabolic acidosis. The absence of blood gas analysis and pH assessment limits the precise definition of metabolic acidosis, as decreased serum bicarbonate levels may also be attributed to respiratory alkalosis in patients with hypobicarbonatemia. Similarly, while a universally accepted definition is still lacking, we have adopted a definition of subclinical metabolic acidosis as a condition where serum bicarbonate levels fall within a borderline range, typically between 22 and 24 mmol/L. Its observational design precludes causal inferences, and residual confounding remains possible despite extensive adjustments for covariates. Important unmeasured factors such as inter-individual differences in ethnicity, genetics, arterial blood gas evaluations, urine studies, as well as dietary intake, hydration status, medication adherence, and sodium bicarbonate dosing were not assessed. Thus, although the analytical approach accounted for potential confounders, the influence of these unmeasured variables cannot be fully excluded. Although we examined a large multicultural cohort across three centers in Toronto, validation in other diverse populations with non-dialysis CKD would be needed to increase the generalizability of these findings. Finally, in restricting our analysis to patients with CKD stage 3–4, our study precludes any inference to the general population or earlier and more advanced stages of CKD.

## Conclusion

Hyperphosphatemia, hypocalcemia, hypovitaminosis D, and hyperparathyroidism—each important features of the CKD-MBD—were prospectively associated with declines in bicarbonate and the development of subclinical and clinical metabolic acidosis in this large multi-ethnic cohort with non-dialysis CKD. Future studies could confirm these associations in other CKD populations, evaluate the effects of acidosis on bone and kidney and other biological mechanisms that might explain these observations, and look for a possible causal association between progressive metabolic acidosis and the development of CKD-MBD.

## Supplementary Information

Below is the link to the electronic supplementary material.

Supplementary file1 (DOCX 181 kb)

## Data Availability

The data collected in both cohorts can be accessed for collaborative research, educational and health equity purposes upon request.
