# Supplementary material for: Prospective associations between the CKD-mineral bone disorder and metabolic acidosis
Source: Int Urol Nephrol. 2025 Dec 18;58(7):2731–9. doi: 10.1007/s11255-025-04902-7 (PMC13309459; doi:10.1007/s11255-025-04902-7)
Supplement: Supplementary file 1 — Supplementary file1 (DOCX 181 kb) [file 11255_2025_4902_MOESM1_ESM.docx]

| 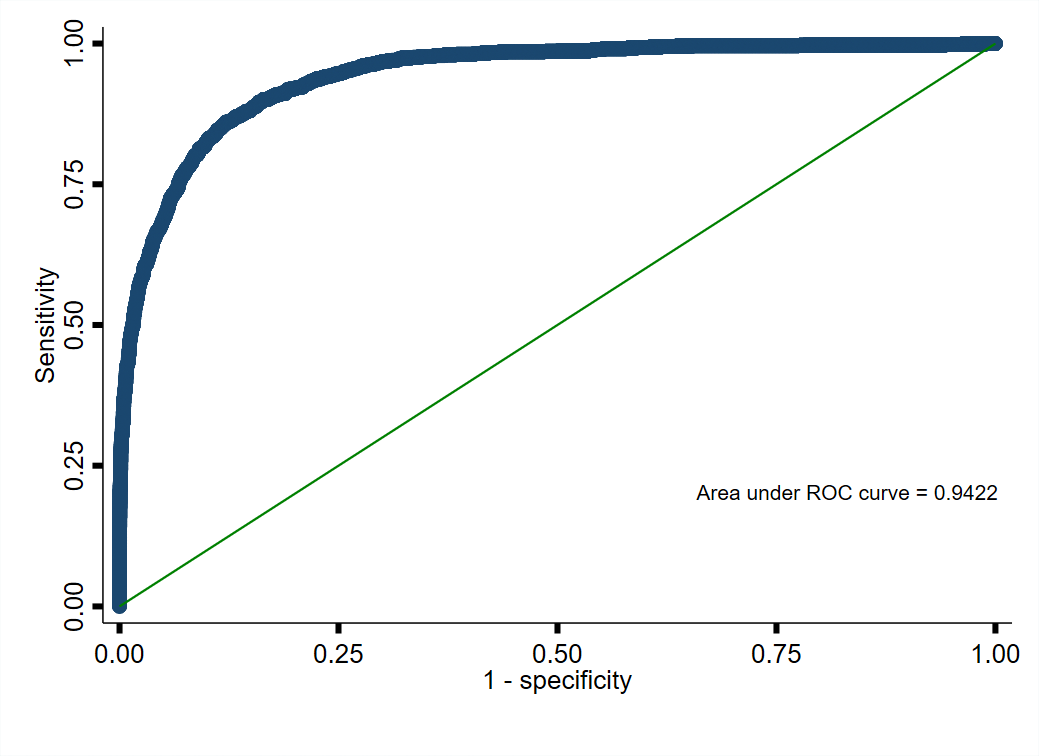 |
| --- |
| Supplemental Figure 1. ROC Curves - Predicting bicarbonate Levels < 22 mmol/L from mixed-effects logistic regression models (Model 2) |

| 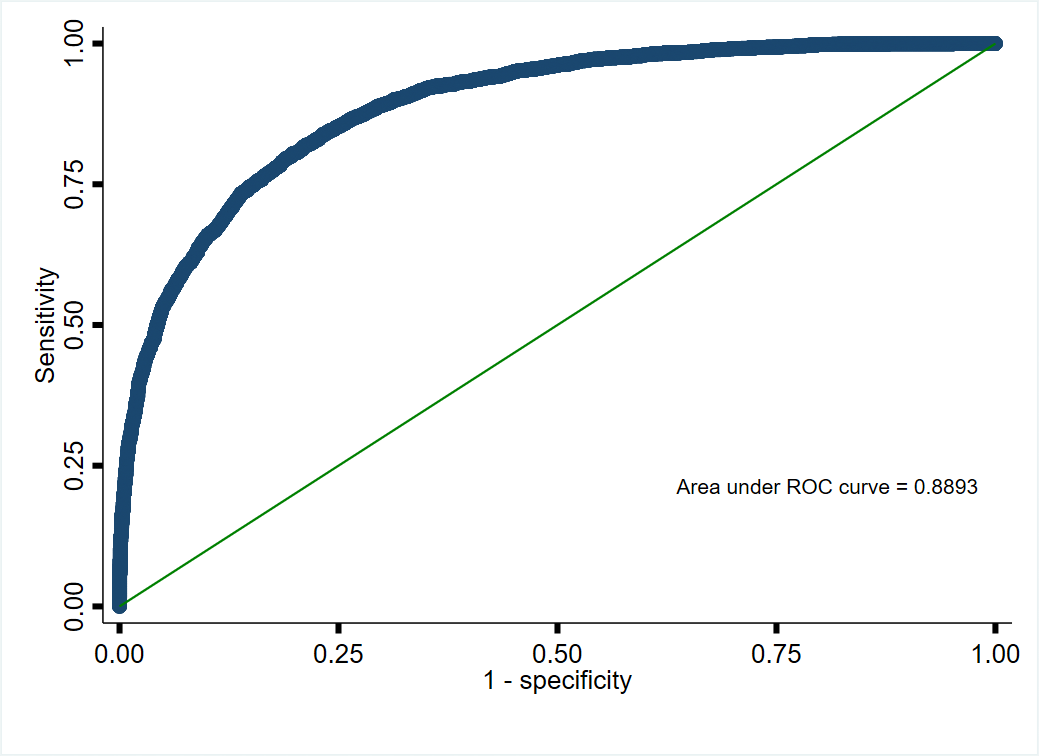 |
| --- |
| Supplemental Figure 2. ROC Curves - Predicting bicarbonate levels between 22-24 mmol/L from mixed-effects logistic regression models. (Model 3) |

Supplemental Table 1: Bootstrap analysis of adjusted changes in CKD-MBD elements in relation to risk for metabolic acidosis

| **Variable** | **Change in plasma**  **bicarbonate** | **Subclinical**  **metabolic acidosis** | **Clinical**  **metabolic acidosis** |
| --- | --- | --- | --- |
|  | per mmol/L, (95% CI), *p* | OR, (95% CI), *p* | OR, (95% CI), *p* |
| **Age (Years)** | 0.02, (0.02, 0.03)  <0.001 | 0.98 (0.97, 0.99)  <0.001 | 0.98 (0.97, 0.99)  <0.001 |
| **Time (per 6 months)** | 0.08, (-0.01, 0.17)  0.080 | 0.94 (0.82, 1.08)  0.358 | 0.78 (0.64, 0.96)  0.020 |
| **Time (non-linear)** | -0.03, (-0.04, -0.02)  <0.001 | 1.03 (1.01, 1.05)  0.001 | 1.04 (1.02, 1.07)  <0.001 |
| **Diabetes Mellitus (Yes)** | -0.39 (-0.54, -0.25)  <0.001 | 1.28 (1.10, 1.49)  0.002 | 1.55 (1.26, 1.92)  <0.001 |
| **Sodium Bicarbonate Use (Yes)** | -0.39, (-0.82, 0.34)  0.421 | 1.28 (0.57, 2.87)  0.547 | 1.63 (0.67, 3.95)  0.282 |
| **eGFR (mL/min/1.73 m²)** | 0.05, (0.04, 0.06)  <0.001 | 0.97 (0.96, 0.98)  <0.001 | 0.95 (0.93, 0.96)  <0.001 |
| **Potassium (mmol/L)** | -0.56, (-0.70, -0.42)  <0.001 | 1.60(1.36, 1.88)  <0.001 | 2.16 (1.76, 2.64)  <0.001 |
| **Log FGF-23** | 0.07, (-0.01, 0.15)  0.097 | 1.06 (0.95, 1.19)  0.317 | 1.02(0.86, 1.22)  0.797 |
| **Log PTH (Spline 1)** | 0.26, (0.03, 0.49)  0.028 | 0.76 (0.56, 1.04)  0.082 | 0.38 (0.40, 0.96)  0.034 |
| **Log PTH (Spline 2)** | -0.25, (-0.52, 0.01)  0.064 | 1.42 (0.98, 2.04)  0.061 | 1.44 (0.86, 2.42)  0.167 |
| **Log 25(OH)D** | 0.38, (0.23, 0.54)   \| <0.001 \| \| --- \| | 0.67(0.55, 0.82)  <0.001 | 0.65 (0.49 0.85)  0.002 |
| **Albumin (g/L)** | -0.07, (-0.09, -0.04)  <0.001 | 1.05 (1.02, 1.08)  0.004 | 1.04 (1.00, 1.09)  0.042 |
| **Phosphate (mmol/L)** | -1.29, (-1.60, -0.98)  <0.001 | 1.92 (1.26, 2.94)  0.003 | 5.03 (3.02, 8.38)  <0.001 |
| **Chloride (mmol/L)** | -0.26, (-0.29, -0.24)  <0.001 | 1.21 (1.18, 1.25)  <0.001 | 1.31 (1.26, 1.37)  <0.001 |
| **Calcium (mmol/L)** | 2.70, (2.09, 3.32)  <0.001 | 0.23 (0.10, 0.52)  <0.001 | 0.15 (0.05, 0.42)  <0.001 |
